# Radiomics Evaluation of Histological Heterogeneity Using Multiscale Textures Derived From 3D Wavelet Transformation of Multispectral Images

**DOI:** 10.3389/fonc.2018.00096

**Published:** 2018-04-04

**Authors:** Ahmad Chaddad, Paul Daniel, Tamim Niazi

**Affiliations:** Division of Radiation Oncology, McGill University, Montreal, QC, Canada

**Keywords:** cancer, discrimination, segmentation, texture, wavelet

## Abstract

**Purpose:**

Colorectal cancer (CRC) is markedly heterogeneous and develops progressively toward malignancy through several stages which include stroma (ST), benign hyperplasia (BH), intraepithelial neoplasia (IN) or precursor cancerous lesion, and carcinoma (CA). Identification of the malignancy stage of CRC pathology tissues (PT) allows the most appropriate therapeutic intervention.

**Methods:**

This study investigates multiscale texture features extracted from CRC pathology sections using 3D wavelet transform (3D-WT) filter. Multiscale features were extracted from digital whole slide images of 39 patients that were segmented in a pre-processing step using an active contour model. The capacity for multiscale texture to compare and classify between PTs was investigated using ANOVA significance test and random forest classifier models, respectively.

**Results:**

12 significant features derived from the multiscale texture (i.e., variance, entropy, and energy) were found to discriminate between CRC grades at a significance value of *p* < 0.01 after correction. Combining multiscale texture features lead to a better predictive capacity compared to prediction models based on individual scale features with an average (±SD) classification accuracy of 93.33 (±3.52)%, sensitivity of 88.33 (± 4.12)%, and specificity of 96.89 (± 3.88)%. Entropy was found to be the best classifier feature across all the PT grades with an average of the area under the curve (AUC) value of 91.17, 94.21, 97.70, 100% for ST, BH, IN, and CA, respectively.

**Conclusion:**

Our results suggest that multiscale texture features based on 3D-WT are sensitive enough to discriminate between CRC grades with the entropy feature, the best predictor of pathology grade.

## Introduction

Colorectal cancer (CRC) is the third most common and newly diagnosed cancer and third most common cause of cancer death in both men and women in the United States (1), accounting for 8% of all new cancer cases per year. An estimated 26, 270 men and 24,040 women died of colorectal carcinoma in 2014 as reported by American Cancer Society (1). The primary treatment modality used is surgical therapy with curative intent, followed by a pathological assessment of the resected tissue which directs subsequent treatments (2).

Colorectal lesions usually exist as a continuum, from benign to malignant forms (3). Most benign lesions slowly start as polyps, i.e., abnormal growths from the inner lining of the intestine that protrude into the intestinal canal. Some of these polyps exhibit abnormal cellular growth and progress into a stage called intraepithelial neoplasia (dysplasia). Intraepithelial neoplasia is a form of pre-cancerous lesion which is highly likely to progress into full-fledged cancer or carcinoma (4).

Initial diagnostic tests include a colonoscopy and biopsy of the abnormal area inside the colon. Histopathological examination of this biopsy confirms whether the lesion is benign, pre-cancerous, or cancerous (5). A gradual, yet distinct continuum of malignancy is found in CRC as a lesion progresses from normal to benign, benign to precancerous, and precancerous to a cancerous lesion. Benign lesions are identified by the increased number of normal cells in an abnormal site, but are incapable of invading nearby tissues or spreading to distant tissues (metastasis). Precancerous lesions or intraepithelial neoplasia are abnormal cells that develop inside and stay within the innermost lining (epithelial) of the colon without growing deeper. Some precancerous lesions grow deeper into the colon (or prostate), crossing the epithelial layer and becoming cancerous. These lesions are graded from well differentiated to moderately and poorly differentiated cancers, depending on their degree of visual similarity or dissimilarity to normal tissue. Cancer development is associated with a disruption in the normal size and shape of cells, number of cells, and the amount of intercellular stroma (ST), which also varies within and between different cancer samples (6).

Pathologists provide diagnoses from biopsy sections (4) by labeling the lesions as benign or cancerous and grading them based on differentiation. Unfortunately, the histological assessment of pathology sections is highly subjective and prone to inter/intra observer variation (2, 7–9), motivating the need for automated or computer-aided diagnosis of pathological slides. Recently, more emphasis has been placed on using digital technologies to produce high-resolution whole slide images (WSI) (10). Texture feature extraction from digital pathology images has been a major focus in the development of computer-aided diagnosis systems (10–12). An early study conducted by Esgiar et al. used correlation and entropy texture features computed from gray-level co-occurrence matrices (GLCM) to differentiate between normal and cancerous tissue (13). They further improved the sensitivity and specificity of classification by incorporating fractal dimensions into the feature analysis (14). Kalkan et al. achieved an accuracy of 75.15% between the four classes of normal, cancerous, adenomatous, and inflammatory colon tissue using 32-bin color channel histograms, GLCM, and structural features (15). Jiao et al. proposed a method for automatic colon cancer detection using GLCM for texture features and support vector machines to achieve a classification accuracy of 96.67% between cancerous and noncancerous images (16). Wavelet features have proven useful in a wide variety of applications, including image compression and preprocessing, and many studies have used texture features based on wavelets for classification (17–19). For example, Hilado et al. used the 2D wavelet transform (2D-WT) to classify whole slide colon cancer images into normal, cancerous, and adenomatous polyp tissue types with 91.11% accuracy (20). However, multiscale texture analysis of pathology tissue (PT) for CRC is still rarely done. For this reason, we broaden our analysis to find scale texture differences between the PT using 3D wavelet transform (3D-WT) filter.

To our knowledge, this is the first work to investigate the link between scale textures in multispectral images and their association with stages of CRC malignancy. Multiscale texture features derived from multispectral images encode thousands of invisible patterns that are complementary to traditional texture based on GLCM, local binary patterns, Laplacian of Gaussian filter, deep learning (10–12, 21), or shape measurements (22). In this context, multiscale texture features measure the heterogeneity in the images of the PT and could be one of the radiomic techniques. Notably, extraction of quantitative imaging features that measure heterogeneity within cancer may be used to guide clinical decisions (23).

Our radiomic analysis approach based on the extraction and analysis of texture features, thus provides an additional means of characterizing the continuum from benign to malignant CRC. This work may lead to a better understanding of the continuum of PT, such as ST, benign hyperplasia (BH), intraepithelial neoplasia (IN) or precursor cancerous lesion, and carcinoma (CA). To conclude, we sought to describe the relationship between multiscale texture features and PT grades.

## Materials and Methods

**Figure 1:**
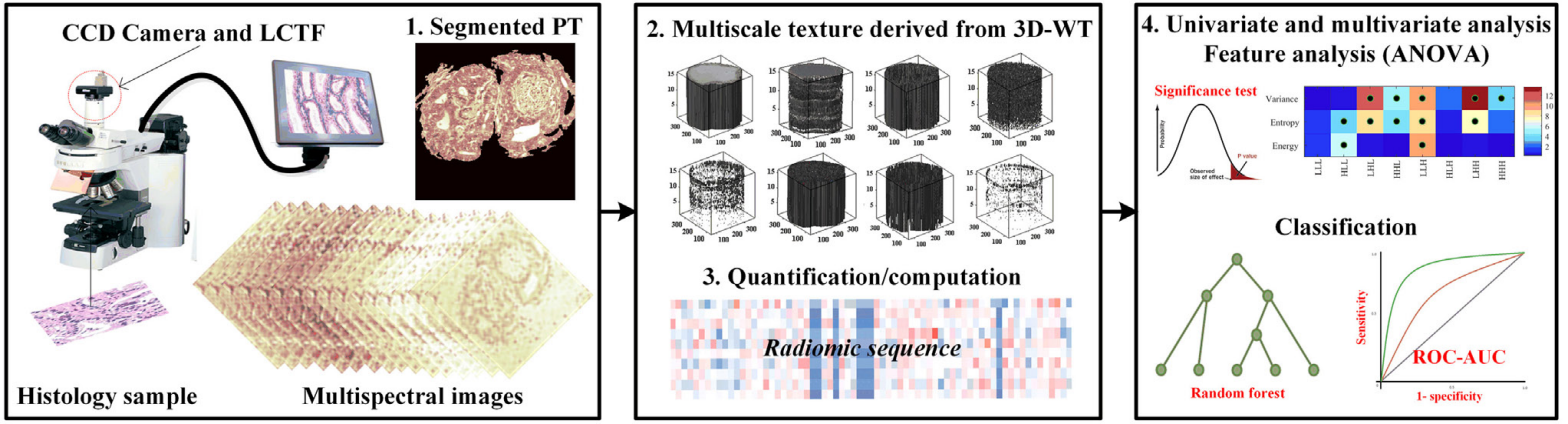
The proposed pipeline for identifying colorectal cancer (CRC) histopathology [i.e., stroma, benign hyperplasia (BH), intraepithelial neoplasia, and carcinoma] using multiscale texture features derived from multi-spectral images. Preprocessing steps consists of biopsy sample preparation, data acquisition from optical microscopy system, then segmentation of sections using the active contour model. Multiscale texture was derived from segmented pathology tissue using 3D-wavelet transform. Texture was quantized by three quantifier functions (i.e., variance, entropy, and energy). Univariate and multivariate analysis was performed using ANOVA significance test and random forest classifier model to distinguish between malignancy grades.

The flowchart explains the proposed work used to evaluate the performance of multiscale textures for continuum CRC (Figure 1). Our analysis pipeline consists of (1) sample preparation and data acquisition derived from an optical microscopy system with charge coupled camera and liquid crystal tuneable bandpass filter that offers multispectral images, (2) quantification of texture-based 3D-WT that represents the quantified features from each of 3D-WT bands of segmented pathology regions, and (3) univariate ANOVA analysis to show significant features in differences between texture of PT. Multivariate analysis based on random forest (RF) classifier model was used to classify between PT. The pipeline is shown in Figure 1, the steps are as follows.

### Sample Preparation and Data Acquisition

**Figure 2:**
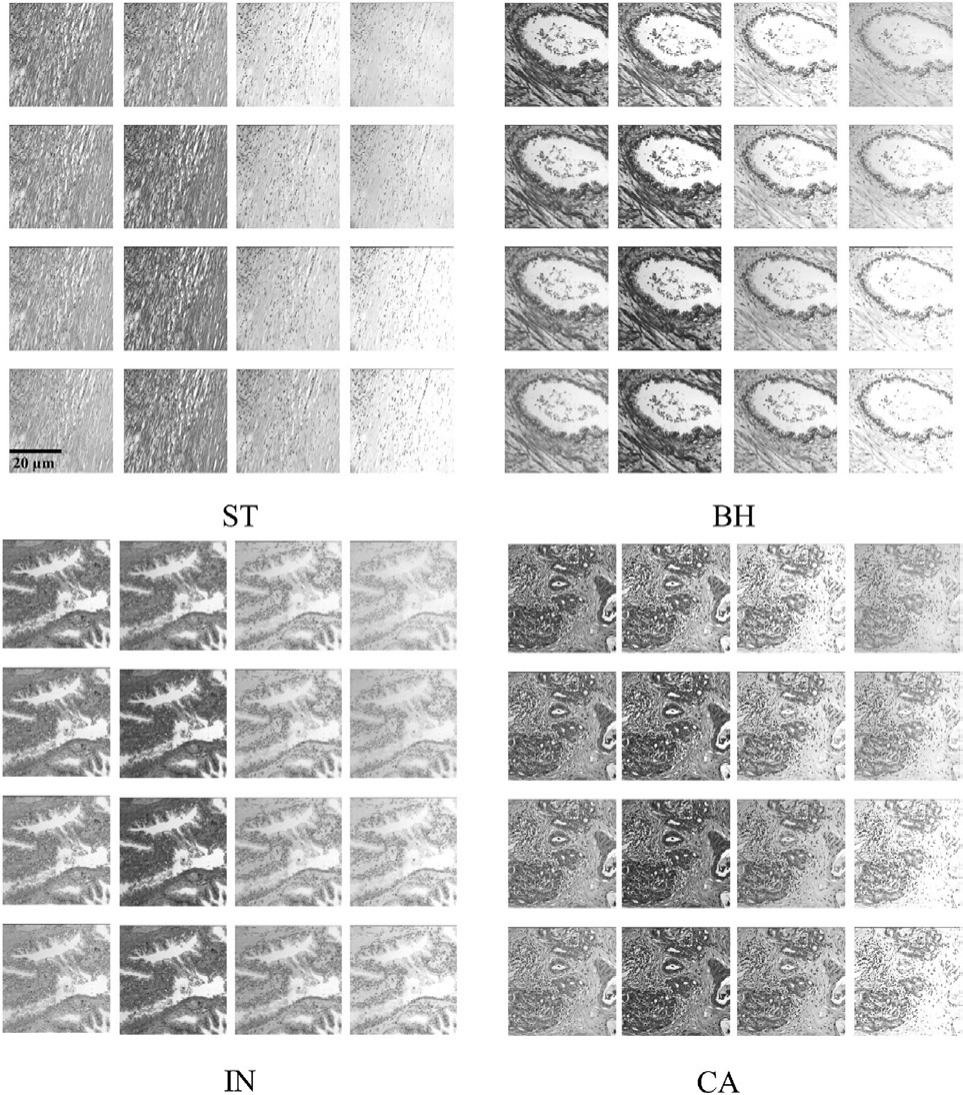
Example of histopathological multispectral images (×40 objective magnification; scale bars = 20 µm) of colorectal cancer samples (ST, stroma; BH, benign hyperplasia; IN, intraepithelial neoplasia; CA, carcinoma). Sixteen multispectral bands were acquired in the wavelength range of 500–650 nm, with 9.375 nm steps between successive bands (23).

We analyzed 3D multispectral digital WSI from 39 patients with colorectal lesions. Whole tissue samples were obtained from intestinal glands with a thickness value of 5 µm and hematoxylin and eosin staining. WSI of biopsy samples obtained using optical microscopy system (Leica TCS NT with Krypton/Argon laser A *Z* series) revealed spatial heterogeneity for each PT type (23). Multispectral digital WSI were obtained from a charge coupled device camera integrated with a liquid crystal tunable filter (LCTF) in an optical microscope system (24). LCTF provides multispectral images of the tissue samples by varying the wavelength of operation (25). In our microscopy system, the LCTF has a bandwidth accuracy of 5 nm, with a controllable wavelength through the visible spectrum range of 500–650 nm. Multispectral images were produced through repeated image capture in various wavelength sub-bands. The LCTF provides 16 multispectral bands across a wavelength range of 500−650 nm with 9.375 nm intervals between successive bands. Thus, each sample consists of a volume of 16 image slices sampled across the wavelength range, providing a rich characterization of abnormal cell type associated with lesions (Figure 2). Note that a colorectal pathologist views images at a magnification of (×40) for labeling PT based on semi-automatic segmentation (22).

### Patients and Dataset

The biopsy samples used in this work were collected in 2009 by the service ANAPAT of the CHU Hospital Brabois with the collaboration of the LCOMS laboratory (http://lcoms.univ-lorraine.fr), France. After excluding samples with incomplete data, a set of 39 patients with colorectal lesions were gathered for a preliminary study without any discrimination based on subject race, gender, and age. Each sample corresponds to one patient, the number of the samples per PT type counts are ST = 9, BH = 10, IN = 9, Ca = 11, where again each data sample consists of 16 multispectral images. The images (i.e., volume images) were normalized by dividing voxel intensity by the SD of the whole volume data to minimize the effects of noise in images (i.e., noise normalization decreases noise) and other external factors (i.e., reduce the variability between images). All the images were reconstructed to a 512 × 512 matrix, where the volume size of 512 × 512 × 16 was taken into consideration in texture feature extraction from 3D-WT of segmented PT.

### Semi-Automatic Segmentation

**Figure 3:**
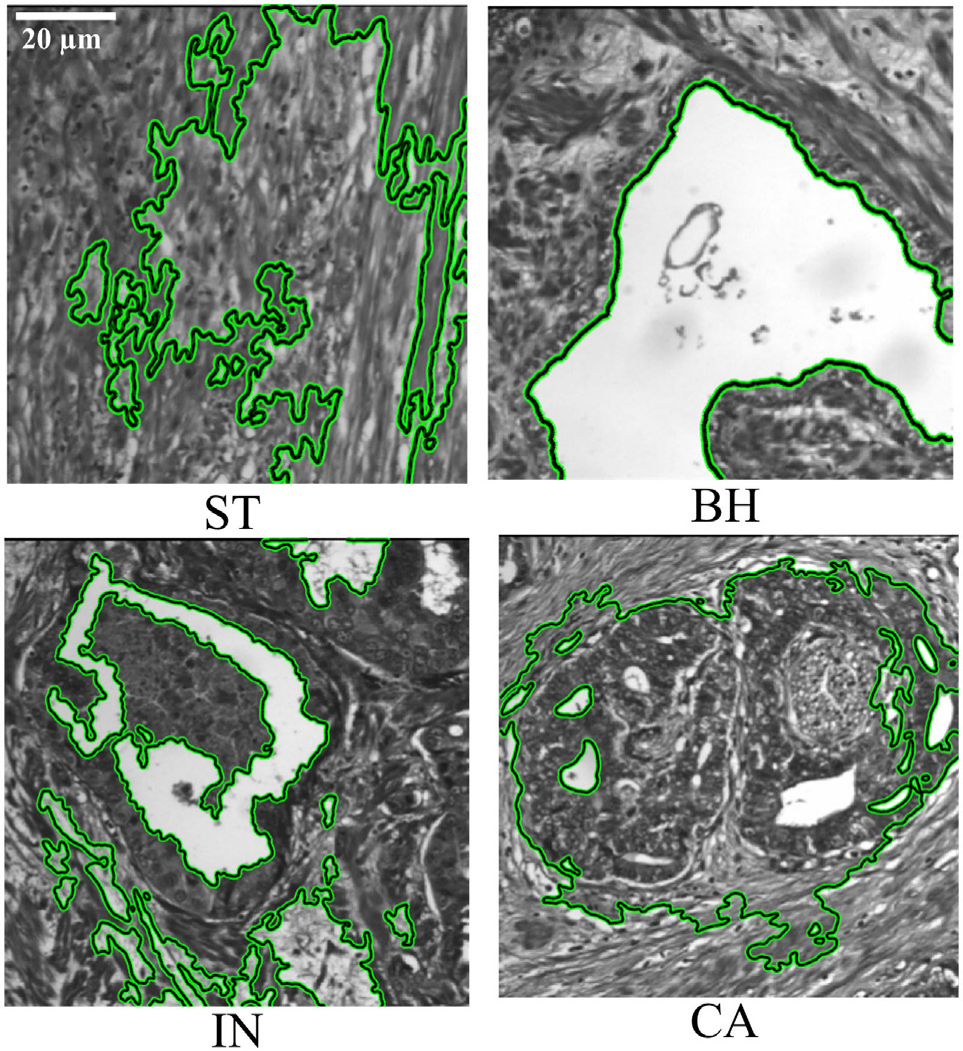
Segmentation of pathology tissue (PTs) by the active contour model applied on histopathological 2D multispectral digital slide images (×40 objective magnification; scale bars = 20 µm) with four PT types (i.e., stroma, benign hyperplasia, intraepithelial neoplasia, carcinoma).

Deformable model-based segmentation is performed by evolving a curve within the image. Active contour is initialized by considering a rectangular contour on the extreme within image. Then for each object detected one contour is automatically drawn surrounding this object. Note that the curve evolution is driven by a combination of external forces, which are computed from the image data, and internal forces which are related to the curve itself. Deformable models using the active contour/snake method have previously been used to detect abnormal tissue types from similar multispectral digital WSI (22), detecting the object contour in an iterative fashion. The computation time was reduced by automatically limiting the number of iterations using empirical calculations. Images were uniformly down-sampled by a factor of 8 in each spatial dimension prior to active contour detection of PT, after which the detected contours were upsampled to the original resolution for analysis. Down-sampling was an important step in decreasing the computation time of active contour detection and had a minimal effect on the final segmentation result. The PT types from WSI were then assessed by a board certified colorectal surgeon (i.e., Figure 3). Figure 3 shows the result of PTs segmented using several steps. The process of PT detection from multispectral images is complicated by digital slide image areas which contain similar ranges of gray-level intensities and irregular shapes. For this reason, our software displays all automatically detected regions of interest and allows a pathologist to select those regions that should be used for the texture analysis. Active contour segmentation accuracy was assessed by comparing against manual segmentation of PT that was done by two pathologists in a blind fashion, and are considered as ground truth. Two similarity measures were used to evaluate segmentation agreement, the Jaccard-similarity-coefficient (JSC) (26), and the dice-similarity-coefficient (*DSC*) (27). The false positive (*FPR*) and false negative rates (*FNR*) were also considered. The *JSC* measures the degree of agreement between manual ground truth and automatic segmentations and is expressed as

(1) $$JSC(A,B)=\frac{\left|A\cap B\right|}{\left|A\cup B\right|}$$

where *A* and *B* are the areas of ground truth and segmented PT, respectively. Note that the *JSC* ranges from 1 for complete overlap and perfect agreement to 0 for no overlap, and thus describes the overall level of similarity between segmentations. *DSC* was also employed and can be expressed according to

(2) $$DSC(A,B)=\frac{2\left|A\cap B\right|}{\left|A\right|+\left|B\right|}$$

where |*A*| and |*B*| represent the number of the pixels in A and B, respectively.

Additionally, *FPR* and *FNR* are used to quantify over and under segmentation, these are calculated according to

(3) $$FPR(A,B)=\frac{\left|B\backslash A\right|}{\left|\bar{A}\right|}$$

(4) $$FNR(A,B)=\frac{\left|A\backslash B\right|}{\left|A\right|}$$

where “\” is the set difference operator. We then applied the 3D-WT to generate the multiscale texture from each of the segmented PT.

### Wavelet Transform-Based Feature

Each of the segmented area was then texturized by the 3D-WT. This technique generally represents an arbitrary function described as a superposition of wavelets, which are generated from a mother wavelet by dilations and translations (28). These translations provide a spatial/frequency representation of the signal. Wavelet coefficients then can be considered to represent the projections of the original signal onto multi-resolution subspaces. In the context of wavelet space decomposition, the 3D-WT can be computed by a tensor product according to the expression

(5) $$\begin{array}{l} V^{3}=\left(L^{x}⊕H^{x}\right)⊗\left(L^{y}⊕H^{y}\right)⊗\left(L^{z}⊕H^{z}\right)\\ \;=L^{x}L^{y}L^{z}⊕L^{x}H^{y}L^{z}⊕H^{x}L^{y}L^{z}⊕H^{x}H^{y}L^{z}\\ \;⊕L^{x}L^{y}H^{z}⊕L^{x}H^{y}H^{z}⊕H^{x}L^{y}H^{z}⊕H^{x}H^{y}H^{z} \end{array}$$

where ⊕ and ⊕ denote a space direct sum and convolution, respectively; *H* and *L* denote high- and low-pass filters; *x, y*, and *z* are 3D coordinates.

**Figure 4:**
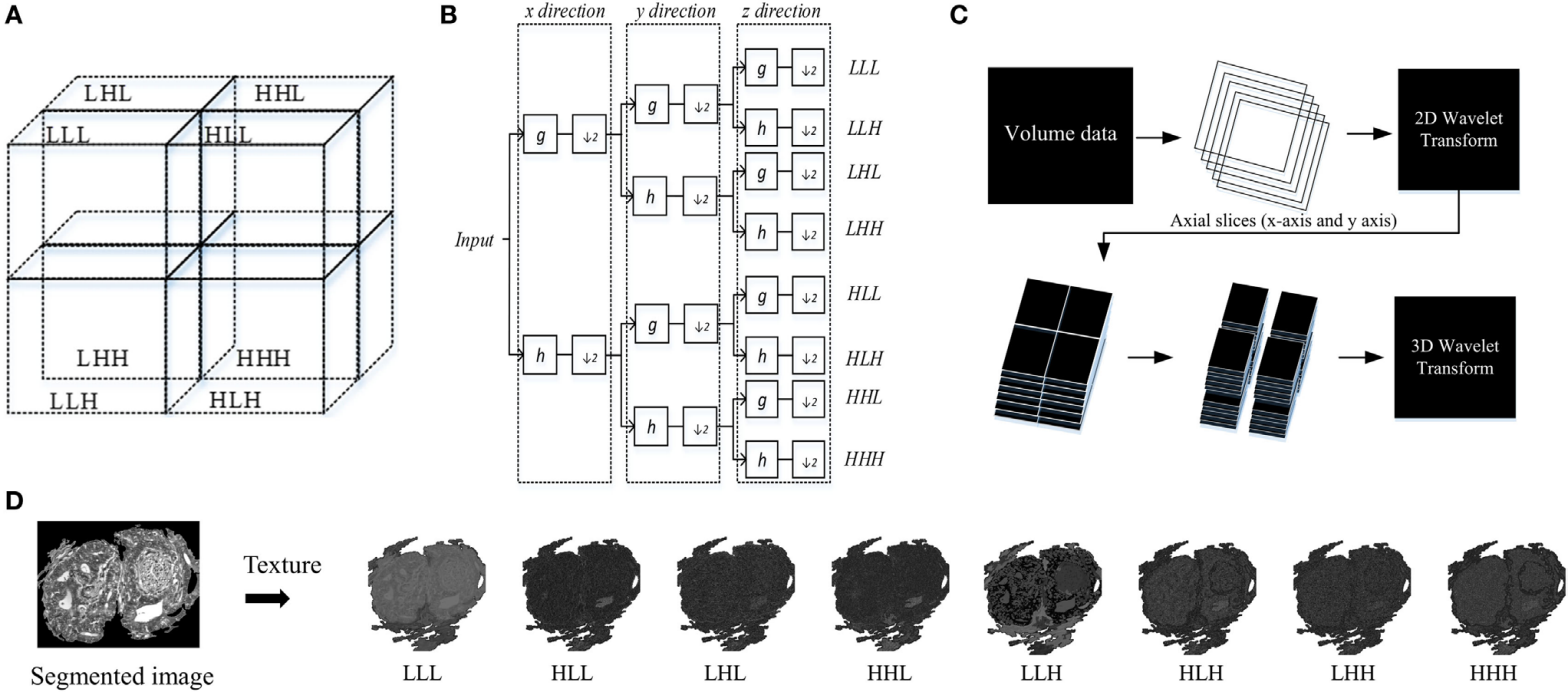
Structure of 3D wavelet decomposition and its corresponding filter bank. **(A)** Schema of sub-band partition of a single level decomposition, **(B)** block diagram of a filter bank for a single level decomposition, **(C)** 3D wavelet transform (3D-WT) scheme based on 2D + 1D model. **(D)** Schema of texture based 3D-WT, an example of carcinoma scheme sample of the eight oct-bands (in this study called multiscale texture) using 3D-WT.

This technique decomposes a 3D-WT space into eight octants, which are called octant sub-bands, and each octant occupies a sub-volume of the 3D wavelet space (29). The multilevel 3D-WT leads to a recursive dyadic 3D sub-band partition based on the low-pass sub-bands, *L^x^L^y^L^z^*. Each of the eight octants can be specified by its directional filters by using *V*^3^ with orientation notations according to the sub-band partition of the 3D-WT (Figure 4). The 3D-WT decomposition is a separable operation, and can thus be computed by applying the 1D dimension wavelet decomposition independently along the *x* and *y* image axes to generate the 2D-WT decomposition, and finally along the *z* axis (Figure 4). In the context of filter banks, 3D wavelet decomposition considers a low-pass filter (*g*) and a high-pass filter (*h*), where the filter coefficients are determined by wavelet basis functions (i.e., Figure 4B). A variety of wavelet types could be used with similar results, in this study we report results using the Daubechies wavelet (db_2_) at one level. The db_2_ wavelet is among the most commonly used and achieves a good spatial-frequency localization trade-off using narrow high-pass and wide low-pass filters (30). A comparison of different wavelet types is out of the scope of this work, and this is left as a future research direction. As our work concerns the multiscale texture analysis of bio-images, the ideal wavelet is tuned to the spatial frequencies characterizing the image textures of interest (28, 31). Asymmetry is an important property of Daubechies wavelets, where the degree of asymmetry increases with the order of the wavelet (32). In previous work, the 3D-WT “2D + 1D” coefficients were used in filtering noise from breast cone-beam computed tomography volumetric data, with two filtering operators using variable coefficients (29). Here, this technique is adapted for 3D-WT texture extraction. Mostly, the fine texture derived from the details (i.e., high-pass filter), while the coarse texture is derived from the approximation (i.e., low-pass filter). Visually, distinct texture scales can be observed from sub-bands (i.e., oct-bands) throughout the 3D-WT applied on CA sample and visualized in 2D and/or 3D images (i.e., Figures 1 and 4D). Moreover, all the functions related to the 3D-WT are available in the *Wavelet Toolbox* as built-in functions in Matlab.

### Texture Quantification

The output textures result of the 3D-WT is a set of eight 3D matrices *V* [*m, n, k*] corresponding to the octant sub-bands (in this study named as multiscale texture), where *m, n*, and *k* are the coordinates along *x, y*, and *z* axis, respectively. These textures are then quantified by three functions, namely, the *Variance, Entropy*, and *Energy*.

The *Variance* function measures how far each texture value in the set from the texture average $\left(\bar{V}\right)$, also it can assess the changes of the WSI heterogeneity. It can be written as follows:

(6) $$Variance=\sum_{m,n,k=1}^{N}{\left|V\left[m,n,k\right]-\bar{V}\right|}^{2}$$

where $\bar{V}=\sum_{m,n,k=1}^{N}V\left[m,n,k\right]$ and *N* is represented the size for a certain band.

The *Entropy* function measures the disorder or complexity of the texture. It reaches a maximum for completely random textures and a minimum for regular textures. Let *Y* be the octant (i.e., sub-band/subvolume) value of a pixel (*x*,*y*) in the segmented region Ω (i.e., ROI). We then discretize the distribution of each sub-band into 256 equal-sized intervals, and denote as Ω*_k_* the subset of pixels within the *k*-th interval. The entropy then can be computed according to

(7) $$\mathrm{Entropy}=-\sum_{k}\frac{\left|\Omega_{k}\right|}{\left|\Omega \right|}\text{log}\frac{\left|\Omega_{k}\right|}{\left|\Omega \right|}$$

Finally, the *Energy* function measures the distribution of energy along the frequency axis over scale and orientation. Energy feature of each sub-band can calculate according to

(8) $$\mathrm{Energy}=\sum_{k}{\left(\frac{\left|\Omega_{k}\right|}{\left|\Omega \right|}\right)}^{2}$$

The functions *Variance, Entropy*, and *Energy* can be collectively used to characterize the structure of information in the textures volumetric WSI data.

### Statistical Analysis and Classification

Each volume sample is represented by eight 3D matrices which correspond to the octant sub-bands. For each sample, we extracted a feature vector of 24 features derived from the eight sub-bands quantified by three functions namely *Variance, Entropy*, and *Energy*. Then, *Z*-score normalization was employed on each of the feature vectors, which converted the features to zero mean and unit variance as follows:

(9) $$r_{n}=\frac{r-mean}{\sigma }$$

where *r* is the original value, *r_n_* is the new value, and the mean and σ are the average and SD of the original data, respectively.

Thereafter, we performed univariate analysis based on ANOVA significant test for comparing between PT and multiscale texture types (i.e., LLL, HLL, LHL, HHL, LLH, HLH, LHH, and HHH). This is a significance test for analyzing experimental data in which one or more response variables are measured under various conditions identified by one or more classification variables (33). We used this test to assess the significance of scale texture features for comparing between PT. To account for these multiple comparisons (e.g., 3 quantifier functions and 8 scale of texture, for a total of 24 tests), *p*-values were corrected following Holm–Bonferroni correction (34) and the statistical significance of features was assessed at *p*-value <0.01.

For multivariate analysis, we considered the RF classifier models to predict the abnormalities tissue types in the WSI. Although various classifiers could be used for this task, we chose the RF as it works well and can be used to inspect the features most dominant in classification (35). A fivefold cross-validation strategy was used to measure the area under the curve (AUC), classifier accuracy, sensitivity, and specificity, where training images are divided into five equal-sized subsets, and in each fold, one subset is put aside for testing and the remaining four subsets are used to train a single RF model, using 100 decision trees. The overall performance of the model was then measured as the average AUC (or classifier accuracy, sensitivity, and specificity) obtained over all fivefolds.

Note that true positive (*TP*) and true negative (*TN*), is the number of positive and negative samples correctly classified. For example, TP of ST is all the ST samples that are classified as ST, TN of ST is all non ST samples that are not classified as ST. False positive (*FP*) and false negative (*FN*) is the numbers of positive and negative samples incorrectly classified (36). For example, FP of ST is all non ST samples that are classified as ST, FN of ST is all ST samples that are not classified as ST. Similar computation was considered for TP, TN, FP and FN of BH, IN, and CA samples.

Then, TP + FN is the total numbers of test samples of the considered class.

*Accuracy* represents the correctly classified samples and can be expressed by

(10) $$\mathrm{Accuracy}=\frac{\text{TP}+\text{TN}}{\text{TP}+\text{FP}+\text{TN}+\text{FN}}$$

*Sensitivity* is a measure of the capability of a classifier to recognize the positive class patterns. It can be expressed according to

(11) $$\mathrm{Sensitivity}=\frac{\text{TP}}{\text{TP}+\text{FN}}$$

*Specificity* is a measure of the capability of a classifier to recognize the negative class patterns. It can be expressed by the following equation:

(12) $$\mathrm{Specificity}=\frac{\text{TN}}{\text{TN}+\text{FP}}$$

All the functions related the statistical analysis is available in the Statistics and Machine Learning Toolbox as built-in functions in Matlab. The source code for the segmentation, wavelet analysis, and statistical analysis is available upon request.

## Results

The proposed texture features extraction using 3D-WT was applied on 39 patients with colorectal lesions. Univariate analysis using ANOVA significance test was used to identify texture features that were significantly different between PT types.

### Segmentation

Active contour segmentation model showed that malignancy types were correctly detected and located with a high performance of similarity metrics. *JSC* shows a similarity range of 75−82% with the best performance achieved with Ca PT. Meanwhile, *DSC* shows a similarity range of 86−89% with the best performance achieved with Ca PT type. These metrics confirmed the feasibility of active contour segmentation method to determine the PT types and specifically the carcinoma lesions (Table 1).

**Table 1:** Average of similarity metrics between ground truth and segmented pathology lesions.

Metrics	Stroma	Benign hyperplasia	Intraepithelial neoplasia	Carcinoma
Jaccard-similarity-coefficient	80	76	75	82
Dice-similarity-coefficient	87	86	86	89
False positive rates	05	07	05	04
False negative rates	15	18	20	14

### Univariate Analysis

**Figure 5:**
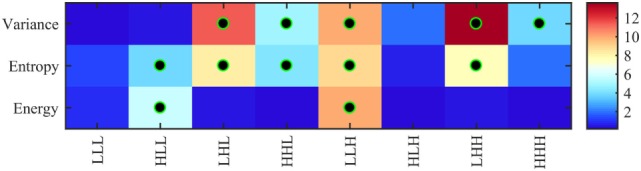
Heatmap of ANOVA test *p-*values (−log_10_ scale) using 24 features (8 variance, 8 entropy, and 8 energy) derived from 3D-wavelet transform bands (LLL, HLL, LHL, HHL, LLH, HLH, LHH, and HHH) comparing the four groups of lesions (stroma, benign hyperplasia, intraepithelial neoplasia, and carcinoma). Features leading to groups with significantly different texture profiles (i.e., corrected *p* < 0.01) are indicated with a black–green circle.

Figure 5 shows the significance of each of the texture features which identify the four pathology lesions (i.e., ST, BH, IN, and CA) from CRC patient biopsies. We found that 12 features were statistically significant (*p* < 0.01) and could identify the four lesions types. These features are derived from multi-scale textures of variance (i.e., five features: LHL, HHL, LLH, LHH, and HHH), entropy (i.e., five features: HLL, LHL, HHL, LLH and HHH), and energy (i.e., two features: HLL and LLH). Notably, features derived from textures of LLL and HLH bands were not significant after multiple corrections. However, in multivariate analysis, the combined features derived from these bands produced a high classifier accuracy. Table 2 shows the average and SD values of each feature across eight 3D-WT band and pathology lesions types.

**Table 2:** Average ± SD for each of features across 3D-WT bands and four pathology tissue (PT) types.

Feature	3D-Wavelet transform band	PT types				*p*
		Stroma	Benign hyperplasia	Intraepithelial neoplasia	Carcinoma	
Variance	LLL	5.463 ± 0.587	6.165 ± 1.411	5.610 ± 1.304	5.948 ± 0.285	0.419
	HLL	3.579 ± 0.339	3.613 ± 1.773	2.826 ± 0.432	3.237 ± 0.597	0.299
	LHL	1.225 ± 0.360	0.650 ± 0.309	0.790 ± 0.542	2.779 ± 0.599	<0.01*
	HHL	2.369 ± 0.457	1.364 ± 0.448	1.649 ± 0.641	3.122 ± 1.094	<0.01*
	LLH	1.776 ± 0.330	1.641 ± 0.653	1.577 ± 0.599	3.557 ± 0.440	<0.01*
	HLH	1.475 ± 0.197	1.264 ± 0.334	1.371 ± 0.424	2.472 ± 1.593	0.014
	LHH	0.813 ± 0.287	0.628 ± 0.320	0.764 ± 0.538	2.745 ± 0.438	<0.01*
	HHH	3.235 ± 0.294	2.401 ± 0.472	2.804 ± 0.614	4.096 ± 1.253	<0.01*

Entropy	LLL	49.892 ± 0.424	50.007 ± 1.620	51.002 ± 0.758	50.691 ± 0.163	0.037
	HLL	41.445 ± 0.252	41.966 ± 1.201	42.201 ± 0.256	40.533 ± 0.842	<0.01*
	LHL	9.339 ± 0.591	7.673 ± 0.849	8.892 ± 0.523	9.822 ± 0.214	<0.01*
	HHL	15.434 ± 0.559	14.602 ± 0.590	15.763 ± 1.078	14.054 ± 0.710	<0.01*
	LLH	5.565 ± 0.485	5.549 ± 0.600	5.920 ± 0.796	7.502 ± 0.242	<0.01*
	HLH	14.618 ± 0.336	14.767 ± 1.677	15.119 ± 0.457	14.135 ± 0.714	0.169
	LHH	7.656 ± 0.526	7.165 ± 0.462	7.708 ± 0.978	9.215 ± 0.346	<0.01*
	HHH	15.896 ± 0.616	15.552 ± 0.345	16.203 ± 1.081	14.841 ± 1.189	0.010

Energy	LLL	15.029 ± 0.288	16.125 ± 1.787	15.861 ± 0.479	15.822 ± 0.230	0.096
	HLL	8.337 ± 0.372	10.224 ± 1.124	8.504 ± 0.447	9.314 ± 0.489	<0.01*
	LHL	0.056 ± 0.033	0.073 ± 0.065	0.061 ± 0.064	0.781 ± 1.840	0.260
	HHL	0.441 ± 0.228	0.741 ± 1.577	0.375 ± 0.180	0.870 ± 1.152	0.662
	LLH	1.392 ± 0.147	1.844 ± 0.622	1.760 ± 0.389	3.535 ± 0.680	<0.01*
	HLH	0.251 ± 0.029	0.871 ± 1.927	0.331 ± 0.133	0.523 ± 0.473	0.549
	LHH	0.048 ± 0.074	0.040 ± 0.093	0.831 ± 1.890	0.256 ± 0.741	0.291
	HHH	0.000 ± 0.000	0.159 ± 0.502	0.186 ± 0.559	0.702 ± 1.742	0.422

*p-value is statistically significant following Holm–Bonferroni correction.

### Multivariate Analysis

**Figure 6:**
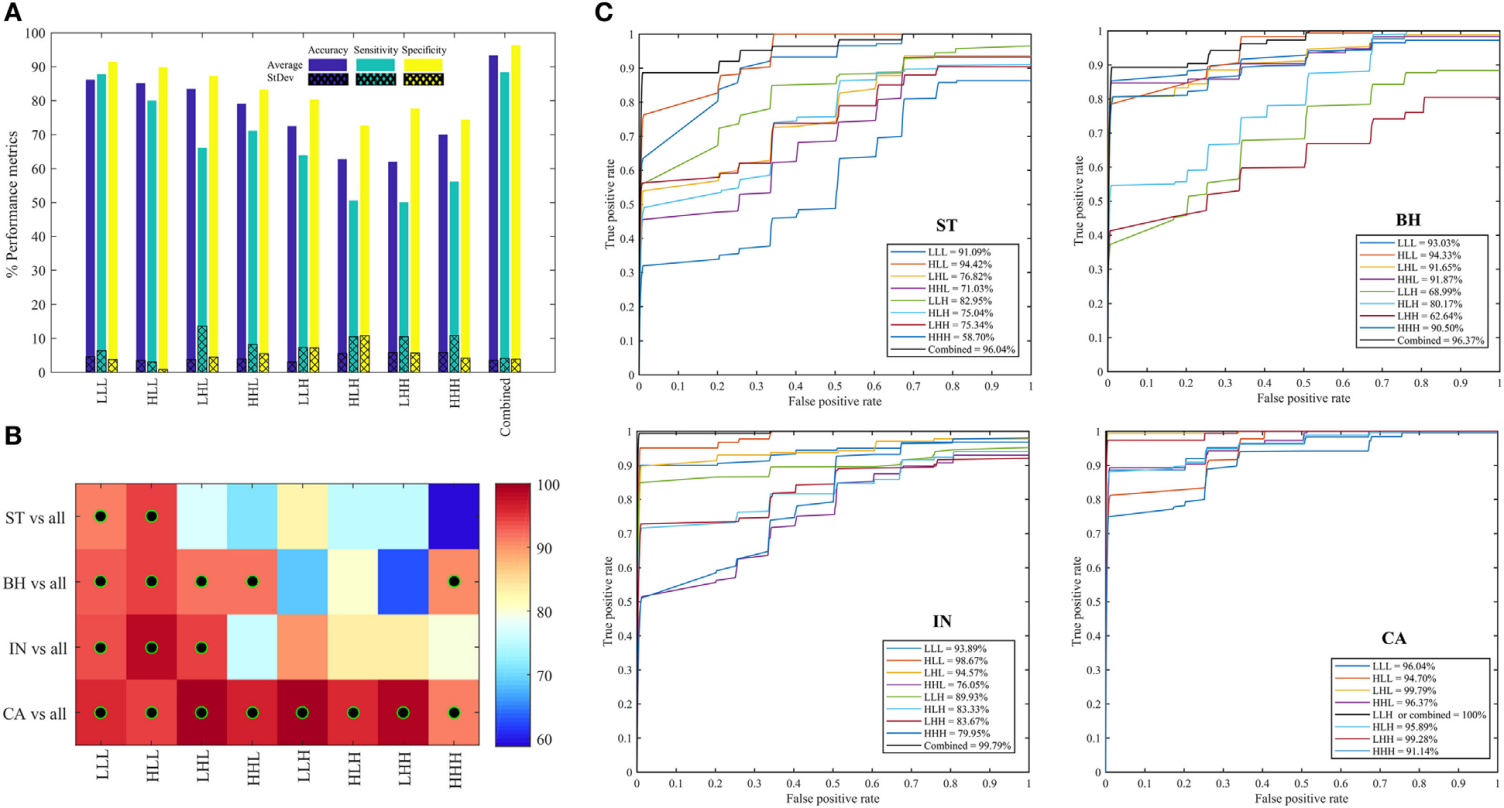
Performance metrics for classifying between stroma, benign hyperplasia, intraepithelial neoplasia, and carcinoma across eight oct-bands derived from 3D-wavelet transform. **(A)** Classifier rate (accuracy, sensitivity, and specificity). **(B)** Heatmap of area under the curve (AUC) values for pathology tissue (PT) in each oct-band, the black–green circle represents the AUC value greater than 90%. **(C)** ROC curves from each PT type across oct-bands.

Figure 6A shows the average (±SD) classifier metrics obtained for each of the eight oct-bands (i.e., texture scale) using three features (variance, entropy, and energy) as input to the classifier model. We found that the combined features derived from all oct-bands achieved the best accuracy, sensitivity and specificity of 93.33 (±3.52), 88.33 (±4.12), and 96.89 (±3.88)%, respectively. We noticed that the texture features derived from LLL, HLL, and LHL provided an average (±SD) range classifier accuracy, sensitivity, and specificity of 83.44–86.16 (±3.4–4.6)%, 66.11–87.78 (±3.0–13.66)%, and 87.28–91.44 (±0.8–4.4)%, respectively. In comparison, weaker performance was achieved when using HHL, LLH, HLH, LHH, and HHH. This demonstrates that the weak discrimination achieved based on univariate analysis (i.e., LLL in ANOVA test) leads to a good classifier metrics when combining features within the RF model. Figures 6B,C shows the area under the ROC curves for each PT type (i.e., ST, BH, IN, and CA) across all the eight oct-bands. We found that the features derived from eight oct-bands of carcinoma tissue achieved an AUC range value of 91.14–100%. In contrast, only five (LLL, HLL, LHL, HHL, and HHH), three (LLL, HLL, and LHL), and two features (LLL and HLL), respectively derived from BH, IN, and CA that are achieved over 90%. We observed that the best AUC values of 96.04, 96.37, 99.79, and 100% were derived from the combined band features in ST, BH, IN, and CA, respectively. Comparing the bands, texture features derived from HLL band achieved the higher AUC value of ST (94.42%), BH (94.33%), and IN (98.67%). However, texture derived from LLH of CA has achieved the best AUC value of 100% (Figure 6C).

**Figure 7:**
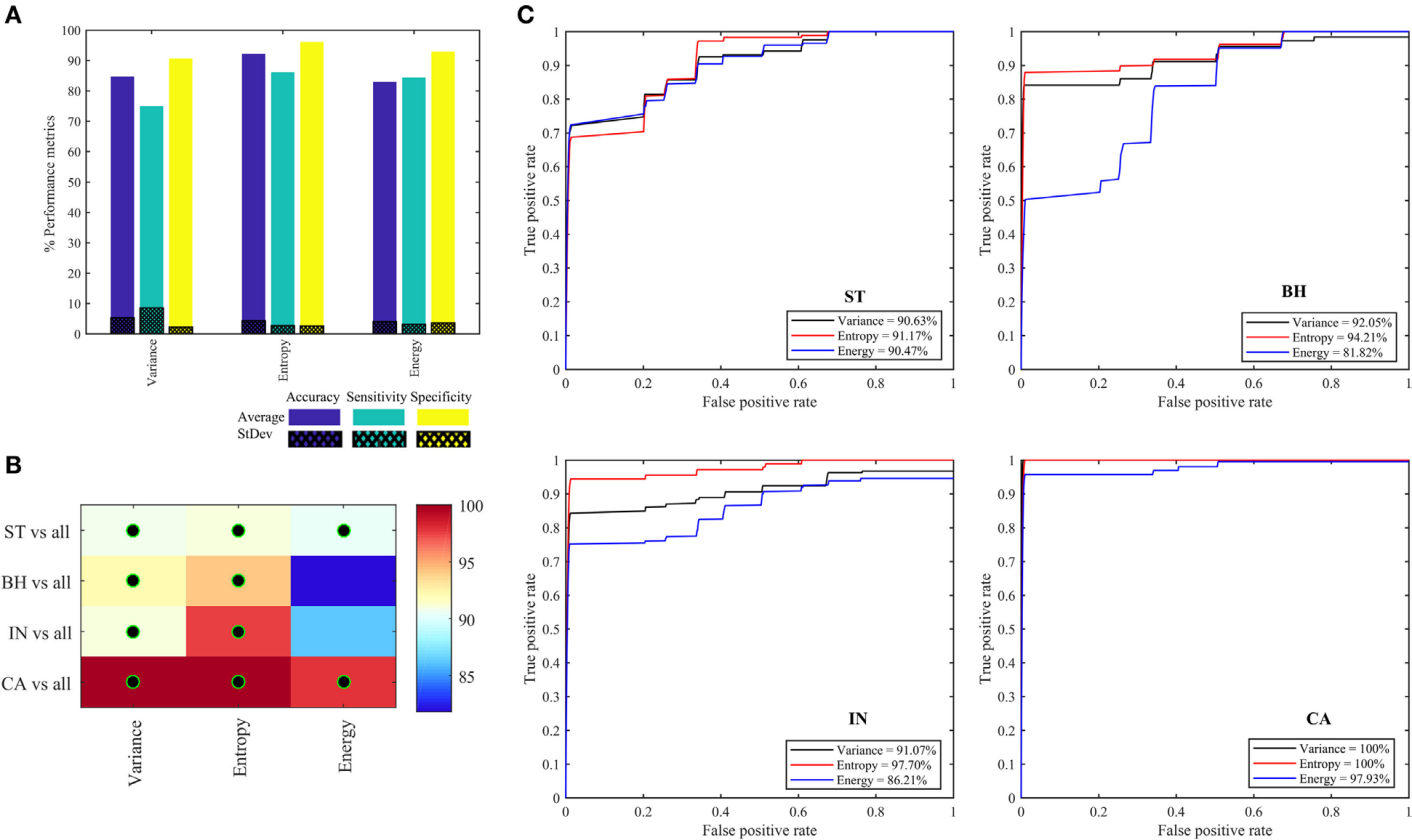
Performance metrics for classifying and predicting stroma, benign hyperplasia, intraepithelial neoplasia, and carcinoma using the features (variance, entropy, and energy) derived from combined eight oct-bands. **(A)** Classifier rate (accuracy, sensitivity, and specificity). **(B)** Heatmap of area under the curve (AUC) values for pathology tissue (PT) based on each feature, black–green circle represents the AUC value greater than 90%. **(C)** ROC curves from each PT type based on three quantifier functions (each quantifier generates eight features).

Figure 7A shows the performance metrics obtained for each of the three quantifier functions (variance, entropy, and energy) derived from eight oct-bands (texture scale) that are combined (LLL, HLL, LHL, HHL, LLH, HLH, LHH, and HHH) and are used as the input to the classifier model. We found that the combined entropy features of eight oct-bands provide a better classifier metrics with an average (±SD) accuracy, sensitivity and specificity of 92.22 (±4.3), 86.11 (±2.7), 96.11 (±2.6)%, respectively, compared to variance (i.e., accuracy of 84.12%, sensitivity of 75.00%, and specificity of 91.33%) and energy features (i.e., accuracy of 83.01%, sensitivity of 84.44%, and specificity of 92.94%). Figures 7B,C shows the area under the ROC curves for all the PT types. We found that ST, BH, IN, and CA achieved, respectively, an average AUC value range of 90.48–91.18, 81.82–94.21, 86.21–97.71, and 97.93–100%. We observed that the CA type has the highest AUC value based on variance and entropy features. We noticed that the entropy is the common feature that achieved the highest AUC value of ST, BH, IN, and CA (i.e., Figure 7C).

Table 3 reports the classifier metrics in terms of accuracy, sensitivity, and specificity, derived from the texture features of GLCM (37) and db_2_ of 2D-WT (38) and 3D-WT. We see that 3D-WT obtain the highest classifier accuracy of 93.33% compared to 89.74 and 84.62% derived from GLCM and 2D-WT features. This confirms that the texture features derived from the 3D-WT provide more patterns to discriminate between PT types.

**Table 3:** Comparative analysis of the proposed model.

Methods	Classifier metrics (%)		
	Accuracy	Sensitivity	Specificity
Gray-level co-occurrence matrices	89.74	77.78	96.67
2D-Wavelet transform (WT)	84.62	66.67	96.67
Proposed model (3D-WT)	93.33	88.33	96.89

## Discussion

There has been an upsurge in studies related to using image texture features or “radiomics” for computer-assisted diagnosis of digital WSI in the recent years (10, 12, 21, 23, 39, 40). In addition to providing diagnostic information, such analysis may also reveal insights into the underlying biology of cancer, making further investigation into radiomic assessment of CRC a priority. Computer-aided accurate diagnosis has an additional benefit in reducing human effort and cost spent in overtreatment and prevents patient morbidity and mortality associated with under diagnosis and under treatment. However, radiomic features most helpful in predicting pathological lesions and malignancy are still unknown.

Previous publications have demonstrated that combined radiomic features can offer good performance for multi-label colon cancer prediction, with a precision of 73.7% (41). However, colon cancer diagnosis and prognosis depends on the ability to discriminate between the distinct malignancy states which can exist (42), oftentimes within an individual tumor section. Several papers have addressed the issue of discriminating between types of pathology for accurate diagnosis of colon cancer. Most have focused on their radiomic heterogeneity and found a correlation between heterogeneity and malignancy (43, 44). Despite this, minimal interrogation of whether radiomic measurements can discriminate between intermediate states of malignancy has limited translation toward clinical practice.

In this study, we utilized 3D-WT texture features to investigate whether radiomic analysis could derive values which can discriminate malignancy within a tissue section. The active contour segmentation technique was used to semi-automatically define areas within each tissue section and report heterogeneity of malignancy (i.e., ST, BH, IN, and CA, Figure 3) before comparing the findings to that from a registered pathologist. We found concordance between our semi-automated methodology and the pathologist report (i.e., Table 1) suggesting that this methodology can successfully define regional heterogeneity in tissue sections. Twelve derived features were found to be differentially enriched in the various malignancy gradings. Areas enriched for carcinoma tissue were most easily defined by our methodology, where they were highly enriched in LHL, HHL, LLH, LHH, and HHH texture features compared to areas of ST, BH, and IN. Similarly, we found that various features derived from entropy and energy features were enriched in benign hypoplasia (energy-HLL) and intraepithelial neoplasia (entropy-HLL/HHL). Combining texture scales achieved the greatest AUC value (i.e., Figure 6) and confidence of CA region identification. This result is consistent with previous studies which considered similar multispectral images using the GLCM and 2D-WT (22, 23). Despite achieving close performance metrics (23), we suggest that the ability for 3D-WT texture features to integrate information from multi-spectral layers to derive radiomic features may increase confidence compared to 2D-WT as feature values are not dependent on single images.

Notably, our model included an option for the pathologist to select regions of interest for further processing, and we suggest that using the implemented active contour model on FPGA technologies as proposed by Chaddad et al. (45) will accelerate the speed of the processing when dealing with thousands of images. In addition, the high accuracy of discrimination achieved using the methodology presented in this manuscript highlights the feasibility of automated assessment of CRC malignancy using digital WSI. In this context, 3D-WT resolves the similarity of patterns observed in the previous studies (12) by comparing the two popular techniques (i.e., 2D-WT and 3D GLCM).

This study offers a simple approach based on texture feature analysis to evaluate the continuum of CRC from benign to malignant by using four PT types which represent the transformation from benign to malignant cancer. Our study should be validated on a bigger dataset to ensure general applicability. Future directives include multiple combinations of texture features in predicting the continuum of CRC. There is also a necessity to increase the realm of diagnosis to include well differentiated, moderately differentiated, and poorly differentiated cancer. Identifying small changes in texture features that could predict the progress or stability of lesions would be a major breakthrough in early diagnosis and management of these lesions. This should trigger further research of image-based quantitative texture features based on 3D-WT in CRC. Given that the reality of CRC is highly heterogeneous between patients, texture feature analysis is a more comprehensive approach to provide a clear categorization of colorectal lesions than the established methods.

## Conclusion

In this paper, a new approach based on 3D-WT texture features for PT classification of CRC is proposed. This study demonstrates that texture feature extraction based on 3D-WT can be a promising technique for mapping colorectal digital slide images. Image processing techniques can be further applied to make the computer-aided diagnosis more robust, which will drive the development of automated systems able to make preliminary diagnosis of tissues to help triage urgent cases. We propose that the methodology described in this manuscript offers added value to diagnostic pipelines at limited additional cost to health care systems and may improve delivery of patient care.

## Author Contributions

AC performed the experiments, analyzed data, designed the experiments. PD and TN reviewed and wrote the final version, and all of the authors gave the final approval of the manuscript.

## Conflict of Interest Statement

The authors declare that the research was conducted in the absence of any commercial or financial relationships that could be construed as a potential conflict of interest.
